# Application of *f*-lacunary statistical convergence to approximation theorems

**DOI:** 10.1186/s13660-018-1871-z

**Published:** 2018-10-11

**Authors:** Vinod K Bhardwaj, Shweta Dhawan

**Affiliations:** 1 Department of Mathematics, Kurukshetra University, Kurukshetra, India; 2 Department of Mathematics, KVA DAV College for Women, Karnal, India

**Keywords:** 40A35, 41A36, 41A25, 47B38, 40A30, Modulus function, Density, Lacunary statistical convergence, Positive linear operator, Korovkin type approximation theorem

## Abstract

The concept of *f*-lacunary statistical convergence which is, in fact, a generalization of lacunary statistical convergence, has been introduced recently by Bhardwaj and Dhawan (Abstr. Appl. Anal. 2016:9365037, 2016). The main object of this paper is to prove Korovkin type approximation theorems using the notion of *f*-lacunary statistical convergence. A relationship between the newly established Korovkin type approximation theorems via *f*-lacunary statistical convergence, the classical Korovkin theorems and their lacunary statistical analogs has been studied. A new concept of *f*-lacunary statistical convergence of degree *β* ($0 < \beta< 1$) has also been introduced, and as an application a corresponding Korovkin type theorem is established.

## Introduction

### Density by moduli and statistical convergence

The idea of statistical convergence, which is, in fact, a generalization of the usual notion of convergence, was first introduced by Fast [14] and Steinhaus [37] independently in 1951 and since then several generalizations and applications of this concept have been investigated by various authors, namely S̆alát [35], Fridy [16], Aizpuru et al. [1], Aktuğlu [2], Gadjiev and Orhan [18], Mursaleen and Alotaibi [28], and many others.

Statistical convergence depends on the natural density of subsets of the set $\mathbb{N} = \{1,2,3,\ldots\}$. The natural density $d(K)$ of a set $K \subseteq\mathbb{N}$ (see [29, Chap. 11]) is defined by

$$ d(K) = \lim_{n \to\infty}\frac{1}{n} \bigl\vert \{k\leq n \colon k \in K\} \bigr\vert , $$

where $\vert \{ k\leq n: k \in K \} \vert $ denotes the number of elements of *K* not exceeding *n*. Obviously, we have $d(K) =0$ if *K* is finite.

#### Definition 1.1

([22])

Let *X* be a normed space. A sequence $(x_{k})$ in *X* is said to be statistically convergent to some $x \in X$, if for each $\epsilon>0$ the set $\{ k \in\mathbb{N} :\|x_{k} - x\|\geq\epsilon \} $ has natural density zero, i.e.,

$$ \lim_{n \to\infty}\frac{1}{n} \bigl\vert \bigl\{ k \leq n : \Vert x_{k} - x \Vert \geq\epsilon \bigr\} \bigr\vert = 0, $$

and we write it as $st-\lim x_{k}=x$.

Recall [25, 34] that a modulus *f* is a function from $\mathbb{R^{+}}$ to $\mathbb{R^{+}}$ such that (i) $f(x) = 0$ if and only if $x = 0$, (ii) $f(x + y) \leq f(x) + f(y)$ for $x \geq0$, $y \geq 0$, (iii) *f* is increasing, (iv) *f* is continuous from the right at 0. If *f*, *g* are moduli and *a*, *b* are positive real numbers, then $f\circ g$, $af+bg$, and $f\vee g$ are moduli. A modulus may be unbounded or bounded. For example, the modulus $f(x) = x^{p}$ where $0 < p \leq1$, is unbounded, but $g(x) = \frac{x}{(1+ x)}$ is bounded. For the work related to sequence spaces defined by a modulus, one may refer to [1, 5–7, 9, 10, 25] and many others.

Aizpuru et al. [1] have recently introduced a new concept of density by moduli, and consequently obtained a new concept of non-matrix convergence, namely, *f*-statistical convergence which is, in fact, a generalization of the concept of statistical convergence and intermediate between the ordinary and statistical convergence. This idea of replacing the natural density with a density by moduli has also been used to study the concepts of *f*-statistical convergence of order *α* [5], *f*-lacunary statistical convergence [6], *f*-statistical boundedness [10], and deferred *f*-statistical convergence [19].

#### Definition 1.2

([1])

For any unbounded modulus *f*, the *f*-density of a set $K \subset \mathbb{N}$ is denoted by $d^{f} (K)$ and is defined by

$$d^{f} (K) = \lim_{n \to\infty} \frac{f ( \vert \{k \leq n: k \in K \} \vert )}{f(n)} $$

whenever this limit exists.

#### Definition 1.3

([1])

Let *f* be an unbounded modulus and *X* be a normed space. A sequence $(x_{k})$ in *X* is said to be *f*-statistically convergent to $x \in X$, if, for each $\varepsilon > 0$,

$$\begin{aligned} &d^{f}\bigl(\bigl\{ k \in\mathbb{N}: \Vert x_{k} - x \Vert \geq\varepsilon\bigr\} \bigr)= 0, \\ &\mbox{i.e., } \lim_{n \to\infty}\frac{1}{f(n)}f \bigl( \bigl\vert \bigl\{ k \leq n : \Vert x_{k} - x \Vert \geq\varepsilon \bigr\} \bigr\vert \bigr)=0, \end{aligned}$$

and we write it as $f-st\lim x_{k} = x$.

#### Remark 1.4

([1])

For any unbounded modulus *f*, every convergent sequence is *f*-statistically convergent which, in turn, is statistically convergent, but not conversely.

By a lacunary sequence $\theta= (k_{r})$; $r = 0,1,2,\ldots$ , where $k_{0} = 0$, we shall mean an increasing sequence of non-negative integers with $k_{r} - k_{r-1} \to \infty$ as $r \to\infty$. The intervals determined by *θ* will be denoted by $I_{r} = ( k_{r-1}, k_{r}]$, and we let $h_{r} = k_{r} - k_{r-1}$. The ratio $k_{r}/k_{r-1}$, which also occurs frequently, will be denoted by $q_{r}$.

Fridy and Orhan [17] introduced the concept of lacunary statistical convergence as follows:

#### Definition 1.5

Let $\theta= (k_{r})$ be a lacunary sequence. A number sequence $(x_{k})$ is said to be lacunary statistically convergent to *l*, or $S_{\theta}$-convergent to *l*, if for each $\varepsilon > 0$,

$$\begin{aligned} \lim_{r \to\infty}\frac{1}{h_{r}} \bigl\vert \bigl\{ k \in I_{r} : \vert x_{k} - l \vert \geq\varepsilon\bigr\} \bigr\vert &=0. \end{aligned}$$

In this case, we write $S_{\theta}-\lim x_{k} = l$.

Quite recently, Bhardwaj and Dhawan [6] have extended the concept of lacunary statistical convergence to that of *f*-lacunary statistical convergence as follows:

#### Definition 1.6

Let *f* be an unbounded modulus and $\theta= (k_{r})$ be a lacunary sequence. A number sequence $(x_{k})$ is said to be *f*-lacunary statistically convergent to *l*, or $S_{\theta}^{f}$-convergent to *l*, if for each $\varepsilon > 0$,

$$\begin{aligned} \lim_{r \to\infty}\frac{1}{f(h_{r})}f \bigl( \bigl\vert \bigl\{ k \in I_{r} : \vert x_{k} - l \vert \geq \varepsilon\bigr\} \bigr\vert \bigr) &=0. \end{aligned}$$

In this case, we write $S_{\theta}^{f}-\lim x_{k} = l$.

An extension of the concepts of lacunary statistical convergence and *f*-lacunary statistical convergence in a more general setting of normed spaces shall be needed in the present work and is given below.

#### Definition 1.7

Let *X* be a normed space and $\theta= (k_{r})$ a lacunary sequence. A sequence $(x_{k})$ in *X* is said to be lacunary statistically convergent to $x \in X$, if, for each $\varepsilon > 0$,

$$\begin{aligned} \lim_{r \to\infty}\frac{1}{h_{r}} \bigl\vert \bigl\{ k \in I_{r} : \Vert x_{k} - x \Vert \geq\varepsilon\bigr\} \bigr\vert &=0. \end{aligned}$$

In this case, we write $S_{\theta}(X)-\lim x_{k} = x$. However, if there is no confusion regarding the scalar- or vector-valued sequences, we may avoid writing *X* explicitly, i.e., we may simply write $S_{\theta}-\lim x_{k} = x$. The set of all *X*-valued lacunary statistically convergent sequences is denoted by $S_{\theta}(X)$.

#### Definition 1.8

Let *f* be an unbounded modulus, *X* a normed space, and $\theta= (k_{r})$ a lacunary sequence. A sequence $(x_{k})$ in *X* is said to be *f*-lacunary statistically convergent to $x \in X$, if, for each $\varepsilon > 0$,

$$\begin{aligned} \lim_{r \to\infty}\frac{1}{f(h_{r})}f\bigl( \bigl\vert \bigl\{ k \in I_{r} : \Vert x_{k} - x \Vert \geq\varepsilon\bigr\} \bigr\vert \bigr) &=0. \end{aligned}$$

In this case, we write $S^{f}_{\theta}(X)-\lim x_{k} = x$, or simply $S^{f}_{\theta}-\lim x_{k} = x$, as mentioned above. The set of all *X*-valued *f*-lacunary statistically convergent sequences is denoted by $S^{f}_{\theta}(X)$.

### Korovkin-type approximation theorems

The theory of approximation is an area of mathematical analysis, which, at its core, is concerned with the approximation of functions by simpler and more easily calculated functions. In the 1950s, the theory of approximation of functions by positive linear operators developed a lot, when Popoviciu [33], Bohman [11] and Korvokin [23, 24] independently discovered a simple and easily applicable criterion to check if a sequence of positive linear operators converges uniformly to the function to be approximated. This criterion says that the necessary and sufficient condition for the uniform convergence of the sequence $(L_{n})$ of positive linear operators to the continuous function *g* on the compact interval $[a,b]$ is the uniform convergence of the sequence $(L_{n}g)$ to *g* for only the three functions $e_{n}(x) = x^{n}, n= 0,1,2$. This classical result of approximation theory is mostly known under the name of Bohman–Korovkin theorem, because Popoviciu’s contribution in [33] remained unknown for a long time.

Due to this classical result, the monomials $e_{n}, n = 0, 1, 2$, play an important role in the approximation theory of linear and positive operators on spaces of continuous functions. These monomials are often called Korovkin test-functions. This elegant and simple result has inspired many mathematicians to extend this result in different directions, generalizing the notion of sequence and considering different spaces. In this way a special branch of approximation theory arose, called Korovkin-type approximation theory. A complete and comprehensive exposure on this topic can be found in [3].

Statistical convergence had not been examined in approximation theory until 2002. Korovkin first and second approximation theorems were first proved via statistical convergence by Gadjiev and Orhan [18] and Duman [13], in 2002 and 2003, respectively. In 2005, Patterson and Savaş [30] proved the first Korovkin approximation theorem via lacunary statistical convergence. It is quite interesting to note that the lacunary statistical analog of the Korovkin second approximation theorem has not been studied so far. Korovkin-type approximation theorems have been studied via various summability methods by many mathematicians. Quite recently Bhardwaj and Dhawan [8] have obtained *f*-statistical analogs of the classical Korovkin first and second approximation theorems. For a detailed account one may refer to [2, 4, 12, 20, 21, 27, 28] where many more references can be found.

### Correct reformulation of the various analogs of the classical Korovkin first theorem

The authors wish to thank Professor F. Altomare for his help in the correct reformulation of the various analogs of the classical Korovkin first theorem.

For a given closed and bounded interval $[a,b]$, we first introduce the following spaces:

$$\begin{aligned} &F_{c}\bigl([a,b]\bigr) =\bigl\{ g : \mathbb{R} \to\mathbb{R} \mid g \text{ is continuous at every point of } [a,b] \bigr\} , \\ &F_{cb}\bigl([a,b]\bigr) =\bigl\{ g : \mathbb{R} \to\mathbb{R} \mid g \text{ is bounded on $\mathbb{R}$ and continuous at every point of } [a,b] \bigr\} , \\ &F_{c}^{*}\bigl([a,b]\bigr) =\bigl\{ g : \mathbb{R} \to\mathbb{R} \mid g \text{ has period }\pi\mbox{ and continuous at every point of } [a,b] \bigr\} , \\ & \begin{aligned} F_{cb}^{*}\bigl([a,b]\bigr) ={}&\bigl\{ g : \mathbb{R} \to\mathbb{R} \mid g \text{ has period }2\pi,\mbox{ bounded on }\mathbb{R}\mbox{ and continuous at every}\\ &\mbox{point of } [a,b] \bigr\} , \end{aligned} \\ &B\bigl([a,b]\bigr) = \bigl\{ g:[a,b] \to\mathbb{R} \mid g \text{ is bounded} \bigr\} . \end{aligned}$$

The space $B([a,b])$ is a Banach space with norm $\|g\|_{B} = \sup_{x \in [a,b]}|g(x)|$, $g \in B([a,b])$.

We also recall [32] that for any linear spaces $X, Y$ of real functions

1. The mapping $L : X \to Y$ is called a linear operator if
  $$\begin{aligned} L(\alpha f+\beta g)= \alpha L(f)+\beta L(g)\quad\text{for } f, g \in X \text{ and }\alpha, \beta\in\mathbb{R}. \end{aligned}$$
2. If $f \geq0$, $f \in X \Longrightarrow Lf\geq0$, then *L* is a positive linear operator.
3. In order to highlight the argument of the function $Lf \in Y$, we use the notation $L(f,x)$.

The classical Korovkin first theorem [24] is stated in Gadjiev and Orhan [18] as follows:

#### Theorem 1.9

*If the sequence of positive linear operators* $L_{n}: C_{M}[a,b] \to B([a,b])$ *satisfies the conditions*

$$\begin{aligned} &\bigl\Vert L_{n}(1,x) - 1 \bigr\Vert _{B} \to0, \quad \textit{as } n \to\infty, \\ &\bigl\Vert L_{n}(t,x) -x \bigr\Vert _{B} \to0, \quad \textit{as } n \to\infty, \\ &\bigl\Vert L_{n}\bigl(t^{2},x\bigr) - x^{2} \bigr\Vert _{B} \to0, \quad\textit{as } n \to\infty, \end{aligned}$$

*then for any function* $g \in C_{M}[a,b]$, *we have*

$$\begin{aligned} \bigl\Vert L_{n}(g,x) - g(x) \bigr\Vert _{B} \to0, \quad\textit{as } n \to\infty, \end{aligned}$$

*where* $C_{M}[a,b]$ *denotes the space of all functions* *g* *which are continuous at every point of the interval* $[a,b]$ *and bounded on the entire line*.

#### Remark 1.10

The space $C_{M}[a,b]$ of Gadjiev and Orhan [18] is essentially the same as the space $F_{cb}([a,b])$ defined above. A new notation for the same space has been introduced for the sake of notational uniformity.

#### Remark 1.11

There is some inaccuracy in the statement of Theorem 1.9 above, as the equations

$$\begin{aligned} &\lim \bigl\Vert L_{n}(t,x) - x \bigr\Vert _{B} = 0, \quad\text{and} \\ &\lim \bigl\Vert L_{n}\bigl(t^{2},x\bigr) - x^{2} \bigr\Vert _{B} = 0, \end{aligned}$$

do not make any sense since the test functions *t* and $t^{2} \notin C_{M}[a,b]$. It is necessary, indeed, to enlarge the domain of positive linear operators $L_{n}$ by considering the linear subspace $D([a,b])$ of $F_{c}([a,b])$ generated by $F_{cb}([a,b])$ and the functions $1, t$ and $t^{2}$. (We could have taken only *t* and $t^{2}$ instead of $1, t$ and $t^{2}$ since the constant function 1 already belongs to $F_{cb}([a,b]$). This has been done just to place the three test functions $1, t$ and $t^{2}$ together). $D([a,b])$ is, surely, the minimal subspace of $F_{c}([a,b])$ on which the linear operators $L_{n}$ must be defined in order to correctly state the result.

From here onwards, $D([a,b])$ will denote the linear subspace of $F_{c}([a,b])$ generated by $F_{cb}([a,b]) \cup\{1, t, t^{2}\}$.

We are now ready to give the correct reformulation of Theorem 1.9 as follows:

#### Theorem 1.12

*If the sequence* $(L_{n})$ *of positive linear operators* $L_{n} : D([a,b]) \to B([a,b])$ *satisfies the conditions*

$$\begin{aligned} &\lim \bigl\Vert L_{n}(1,x) - 1 \bigr\Vert _{B} = 0, \\ &\lim \bigl\Vert L_{n}(t,x) - x \bigr\Vert _{B} = 0, \\ &\lim \bigl\Vert L_{n}\bigl(t^{2},x\bigr) - x^{2} \bigr\Vert _{B} = 0, \end{aligned}$$

*then for any function* $g \in F_{cb}([a,b])$, *we have*

$$ \lim \bigl\Vert L_{n}(g,x) - g(x) \bigr\Vert _{B} = 0. $$

In the same paper [18], Gadjiev and Orhan have given the statistical analog of Korovkin first theorem as follows:

#### Theorem 1.13

*If the sequence of positive linear operators* $L_{n}: C_{M}[a,b] \to B([a,b])$ *satisfies the conditions*

$$\begin{aligned} &st-\lim \bigl\Vert L_{n}(1,x) - 1 \bigr\Vert _{B} = 0, \\ &st-\lim \bigl\Vert L_{n}(t,x) -x \bigr\Vert _{B} = 0, \\ &st-\lim \bigl\Vert L_{n}\bigl(t^{2},x\bigr) - x^{2} \bigr\Vert _{B} = 0, \end{aligned}$$

*then for any function* $g \in C_{M}[a,b]$, *we have*

$$\begin{aligned} st-\lim \bigl\Vert L_{n}(g,x) - g(x) \bigr\Vert _{B} = 0. \end{aligned}$$

#### Remark 1.14

The same inaccuracy gets repeated in the statement of Theorem 1.13. The corrected version is as follows:

#### Theorem 1.15

*If the sequence* $(L_{n})$ *of positive linear operators* $L_{n} : D([a,b]) \to B([a,b])$ *satisfies the conditions*

$$\begin{aligned} &st-\lim \bigl\Vert L_{n}(1,x) - 1 \bigr\Vert _{B} = 0, \\ &st-\lim \bigl\Vert L_{n}(t,x) - x \bigr\Vert _{B} = 0, \\ &st-\lim \bigl\Vert L_{n}\bigl(t^{2},x\bigr) - x^{2} \bigr\Vert _{B} = 0, \end{aligned}$$

*then for any function* $g \in F_{cb}([a,b])$, *we have*

$$ st-\lim \bigl\Vert L_{n}(g,x) - g(x) \bigr\Vert _{B} = 0. $$

Patterson and Savaş [30] have given the lacunary statistical analog of Korovkin first theorem as follows:

#### Theorem 1.16

*If the sequence of positive linear operators* $L_{n}: C_{M}[a,b] \to B([a,b])$ *satisfies the conditions*

$$\begin{aligned} &S_{\theta}-\lim \bigl\Vert L_{n}(1,x) - 1 \bigr\Vert _{B} = 0, \\ &S_{\theta}-\lim \bigl\Vert L_{n}(t,x) -x \bigr\Vert _{B} = 0, \\ &S_{\theta}-\lim \bigl\Vert L_{n}\bigl(t^{2},x\bigr) - x^{2} \bigr\Vert _{B} = 0, \end{aligned}$$

*then for any function* $g \in C_{M}[a,b]$, *we have*

$$\begin{aligned} S_{\theta}-\lim \bigl\Vert L_{n}(g,x) - g(x) \bigr\Vert _{B} = 0. \end{aligned}$$

#### Remark 1.17

The same inaccuracy gets repeated here also. The corrected version is as follows:

#### Theorem 1.18

*If the sequence* $(L_{n})$ *of positive linear operators* $L_{n} : D([a,b]) \to B([a,b])$ *satisfies the conditions*

$$\begin{aligned} &S_{\theta}-\lim \bigl\Vert L_{n}(1,x) - 1 \bigr\Vert _{B} = 0, \\ &S_{\theta}-\lim \bigl\Vert L_{n}(t,x) - x \bigr\Vert _{B} = 0, \\ &S_{\theta}-\lim \bigl\Vert L_{n}\bigl(t^{2},x\bigr) - x^{2} \bigr\Vert _{B} = 0, \end{aligned}$$

*then for any function* $g \in F_{cb}([a,b])$, *we have*

$$ S_{\theta}-\lim \bigl\Vert L_{n}(g,x) - g(x) \bigr\Vert _{B} = 0. $$

We conclude this section by stating the recently obtained (see [8]) *f*-statistical analogs of the Korovkin first and second approximation theorems as we shall be needing them later in this paper.

#### Theorem 1.19

*Let* *f* *be an unbounded modulus and* $(L_{n})$ *a sequence of positive linear operators* $L_{n} : D([a,b]) \to B([a,b])$. *Then*, *for all* $g \in D([a,b])$,

$$ f-st\lim \bigl\Vert L_{n}(g,x) - g(x) \bigr\Vert _{B} = 0 $$

*if and only if*

$$\begin{aligned} &f-st\lim \bigl\Vert L_{n}(1,x) - 1 \bigr\Vert _{B} = 0, \\ &f-st\lim \bigl\Vert L_{n}(t,x) - x \bigr\Vert _{B} = 0, \\ &f-st\lim \bigl\Vert L_{n}\bigl(t^{2},x\bigr) - x^{2} \bigr\Vert _{B} = 0 . \end{aligned}$$

We shall denote by $D^{*}([a,b])$ the linear subspace of $F_{c}^{*}([a,b])$ generated by $F_{cb}^{*}([a,b])$ and the functions $1, \cos t, \sin t$.

#### Theorem 1.20

*Let* *f* *be an unbounded modulus and* $(L_{n})$ *a sequence of positive linear operators* $L_{n} : D^{*}([a,b]) \to B([a,b])$. *Then*, *for all* $g \in D^{*}([a,b])$,

$$ f-st\lim \bigl\Vert L_{n}(g,x) - g(x) \bigr\Vert _{B} = 0 $$

*if and only if*

$$\begin{aligned} &f-st\lim \bigl\Vert L_{n}(1,x) - 1 \bigr\Vert _{B} = 0, \\ &f-st\lim \bigl\Vert L_{n}(\cos t,x) - \cos x \bigr\Vert _{B} = 0, \\ &f-st\lim \bigl\Vert L_{n}(\sin t,x) - \sin x \bigr\Vert _{B} = 0 . \end{aligned}$$

### Discussion of the main problem

In this paper we mainly prove Korovkin-type approximation theorems via *f*-lacunary statistical convergence. The lacunary statistical analog of the Korovkin second approximation theorem is obtained as a particular case. A relationship between the newly established Korovkin type approximation theorems via *f*-lacunary statistical convergence, the classical Korovkin theorems and their lacunary statistical analogs has been studied. In addition, we also establish a relationship between the *f*-lacunary statistical analogs and *f*-statistical analogs of classical Korovkin first and second approximation theorems. The proofs of our main results, i.e., Theorems 2.3 and 2.13, may appear to contain same calculations from the corresponding old ones but, in fact, there are certain gaps and mistakes in the corresponding earlier published proofs which have been filled in and corrected.

## Main results

### *f*-lacunary statistical analog of the Korovkin first theorem

In order to prove an *f*-lacunary statistical analog of Korovkin first theorem, we need the following lemma.

#### Lemma 2.1

([24])

*If a function* $f : \mathbb{R} \to\mathbb{R}$ *is continuous in the interval* $[a,b]$, *continuous on the right at the point* *b* *and on the left at the point* *a*, *then we can find a* $\delta>0$ *for* $\epsilon>0$ *such that the inequality*

$$ \bigl\vert f(y) - f(x) \bigr\vert < \epsilon $$

*is true if* $|y-x| < \delta$, $a \leq x \leq b$.

#### Remark 2.2

In the above lemma, there is an inaccuracy in the sense that, when Korovkin assumes that “*f* is continuous in the interval $[a, b]$”, then this means for him that $f |[a,b]$ is continuous. For these reasons he adds the additional hypotheses that *f* is continuous on the right at the point *b* and that *f* is continuous on the left at the point *a*. According to the modern terminology, when we assume that $f \in F_{c}([a, b])$, then *f* is continuous at every point of $[a, b]$ and, hence, in particular at *a* (both on the right and on the left) as well as at *b* (both on the right and on the left). Therefore, in the statement of Theorem 1.13 above, due to Gadjiev and Orhan [18], it is correctly stated that formula $st-\lim\|L_{n}(g,x) - g(x)\|_{B} = 0$ holds for every $g \in C_{M}[a,b]$ (*i.e.*, $F_{cb}([a, b]) $) because, for such functions Lemma 2.1 can be applied.

We are now in a position to state and prove the promised *f*-lacunary statistical analog of the Korovkin first theorem.

#### Theorem 2.3

*Let* *f* *be an unbounded modulus*, $\theta= (k_{r})$ *a lacunary sequence and* $(L_{n})$ *a sequence of positive linear operators* $L_{n} : D([a,b]) \to B([a,b])$. *Then*, *for all* $g \in D([a,b])$,

2.1 $$ S^{f}_{\theta}-\lim \bigl\Vert L_{n}(g,x) - g(x) \bigr\Vert _{B} = 0 $$

*if and only if*

2.2 $$\begin{aligned} &S^{f}_{\theta}-\lim \bigl\Vert L_{n}(1,x) - 1 \bigr\Vert _{B} = 0, \end{aligned}$$

2.3 $$\begin{aligned} &S^{f}_{\theta}-\lim \bigl\Vert L_{n}(t,x) - x \bigr\Vert _{B} = 0, \end{aligned}$$

2.4 $$\begin{aligned} &S^{f}_{\theta}-\lim \bigl\Vert L_{n} \bigl(t^{2},x\bigr) - x^{2} \bigr\Vert _{B} = 0 . \end{aligned}$$

#### Proof

Since each of $1, t, t^{2}$ belongs to $D([a,b])$, conditions (2.2)–(2.4) follow immediately from (2.1). Now, let conditions (2.2)–(2.4) hold. To prove (2.1), we first prove that for any $g' \in F_{cb}([a,b])$,

$$ S^{f}_{\theta}-\lim \bigl\Vert L_{n} \bigl(g',x\bigr) - g'(x) \bigr\Vert _{B} = 0. $$

We follow the proof of Theorem 1 of Korovkin [24] up to a certain stage. Since the function $g'$ is bounded on the whole real axis, we can write

2.5 $$ \bigl\vert g'(t) - g'(x) \bigr\vert < 2M, \quad-\infty< t, x < \infty. $$

Further, in view of Lemma 2.1, there exists a $\delta> 0$ for each $\epsilon> 0$ such that

2.6 $$ \bigl\vert g'(t) - g'(x) \bigr\vert < \epsilon\quad\text{for } a\leq x \leq b, \vert t-x \vert < \delta. $$

Putting $\psi(t) = (t-x)^{2}$ (*x* an arbitrary but fixed number in the interval $[a,b]$) and using inequalities (2.5) and (2.6), we have

$$ \bigl\vert g'(t) - g'(x) \bigr\vert < \epsilon+ \frac{2M}{\delta^{2}}\psi(t) \quad\text{for all } t \in(-\infty, \infty). $$

This means

2.7 $$ -\epsilon- \frac{2M}{\delta^{2}}\psi(t) < g'(t) - g'(x) < \epsilon+ \frac{2M}{\delta^{2}}\psi(t) \quad\text{for all } t \in(-\infty, \infty). $$

In fact, if $|t-x|< \delta$, then (2.6) implies (2.7) since $\psi(t) = (t-x)^{2} \geq0$, and if $|t-x|\geq\delta$, then

$$ \frac{2M}{\delta^{2}}\psi(t) \geq\frac{2M}{\delta^{2}}\delta^{2} = 2M, $$

and (2.7) follows from (2.5) since $\epsilon>0$.

In view of monotonicity and linearity of the operators $L_{n}(g',x)$, inequality (2.7) implies

$$ -\epsilon L_{n}(1,x) - \frac{2M}{\delta^{2}}L_{n}(\psi,x) \leq L_{n}\bigl(g',x\bigr) - L_{n} \bigl(g'(x), x\bigr) \leq\epsilon L_{n}(1,x) + \frac{2M}{\delta^{2}}L_{n}(\psi,x). $$

Note that *x* is fixed and so $g'(x)$ is a constant number. Therefore,

2.8 $$ -\epsilon L_{n}(1,x) - \frac{2M}{\delta^{2}}L_{n}( \psi,x) \leq L_{n}\bigl(g',x\bigr) - g'(x)L_{n}(1, x) \leq\epsilon L_{n}(1,x) + \frac{2M}{\delta^{2}}L_{n}(\psi,x). $$

But

2.9 $$ L_{n}\bigl(g',x\bigr) - g'(x) = \bigl[L_{n}\bigl(g',x\bigr) - g'(x)L_{n}(1, x) \bigr] + g'(x) \bigl[L_{n}(1, x) - 1\bigr]. $$

Using (2.8) and (2.9), we have

2.10 $$ L_{n}\bigl(g',x\bigr) - g'(x) \leq\epsilon L_{n}(1,x) + \frac{2M}{\delta^{2}}L_{n}(\psi ,x) + g'(x) \bigl[L_{n}(1, x) - 1\bigr]. $$

Now, let us estimate $L_{n}(\psi,x)$ as follows:

2.11 $$\begin{aligned} L_{n}(\psi,x) &= L_{n}\bigl(t^{2}-2tx+x^{2}, x\bigr) \\ &= L_{n}\bigl(t^{2},x\bigr) - 2xL_{n}(t,x)+x^{2}L_{n}(1,x) \\ &=\bigl[L_{n}\bigl(t^{2}, x\bigr) - x^{2}\bigr]- 2x\bigl[L_{n}(t, x) - x\bigr]+ x^{2}\bigl[L_{n}(1, x) - 1\bigr]. \end{aligned}$$

Using (2.11) in (2.10), we have

$$\begin{aligned} &L_{n}\bigl(g',x\bigr) - g'(x) \\ &\quad \leq\epsilon L_{n}(1,x) + \frac{2M}{\delta^{2}} \bigl\{ \bigl[L_{n}\bigl(t^{2}, x\bigr) - x^{2}\bigr]- 2x \bigl[L_{n}(t, x) - x\bigr]+ x^{2}\bigl[L_{n}(1, x) - 1\bigr] \bigr\} \\ &\qquad {}+ g'(x) \bigl[L_{n}(1, x) - 1\bigr] \\ &\quad = \biggl(\epsilon+\frac{2M}{\delta^{2}}x^{2}+g'(x) \biggr) \bigl[L_{n}(1, x) - 1\bigr]+ \frac{2M}{\delta^{2}}\bigl[L_{n} \bigl(t^{2}, x\bigr) - x^{2}\bigr]-\frac{4M}{\delta^{2}}x \bigl[L_{n}(t, x) - x\bigr] +\epsilon. \end{aligned}$$

Therefore,

2.12 $$\begin{aligned} & \bigl\Vert L_{n}\bigl(g',x\bigr) - g'(x) \bigr\Vert _{B} \\ &\quad \leq \biggl(\epsilon+\frac{2M}{\delta^{2}}k_{1}+M \biggr) \bigl\Vert L_{n}(1, x) - 1 \bigr\Vert _{B} + \frac{2M}{\delta^{2}} \bigl\Vert L_{n}\bigl(t^{2}, x\bigr) - x^{2} \bigr\Vert _{B} \\ &\qquad {}+ \frac{4M}{\delta ^{2}}k_{2} \bigl\Vert L_{n}(t, x) - x \bigr\Vert _{B} + \epsilon \\ &\quad \leq K \bigl( \bigl\Vert L_{n}(1, x) - 1 \bigr\Vert _{B}+ \bigl\Vert L_{n}(t, x) - x \bigr\Vert _{B}+ \bigl\Vert L_{n}\bigl(t^{2}, x\bigr) - x^{2} \bigr\Vert _{B} \bigr) +\epsilon, \end{aligned}$$

where $k_{1}= \max\{a^{2},b^{2}\}, k_{2} = \max\{|a|,|b|\}$ and $K = \max \{\epsilon+\frac{2M}{\delta^{2}}k_{1}+M, \frac{4M}{\delta^{2}}k_{2}, \frac {2M}{\delta^{2}} \}$. For any $\epsilon'>0$, choose $\epsilon>0$ such that $\epsilon<\epsilon '$. Now, from inequality (2.12), we have

2.13 $$\begin{aligned} & \bigl\vert \bigl\{ n \in I_{r} : \bigl\Vert L_{n} \bigl(g',x\bigr) - g'(x) \bigr\Vert _{B} \geq\epsilon' \bigr\} \bigr\vert \\ &\quad \leq \biggl\vert \biggl\{ n \in I_{r} : \bigl\Vert L_{n}(1, x) - 1 \bigr\Vert _{B}+ \bigl\Vert L_{n}(t, x) - x \bigr\Vert _{B}+ \bigl\Vert L_{n}\bigl(t^{2}, x\bigr) - x^{2} \bigr\Vert _{B} \geq\frac{\epsilon'-\epsilon}{K} \biggr\} \biggr\vert . \end{aligned}$$

Now, write

$$\begin{aligned} &D:= \biggl\{ n \in I_{r} : \bigl\Vert L_{n}(1, x) - 1 \bigr\Vert _{B}+ \bigl\Vert L_{n}(t, x) - x \bigr\Vert _{B}+ \bigl\Vert L_{n}\bigl(t^{2}, x\bigr) - x^{2} \bigr\Vert _{B} \geq\frac{\epsilon'-\epsilon}{K} \biggr\} , \\ &D _{1}:= \biggl\{ n \in I_{r} : \bigl\Vert L_{n}(1, x) - 1 \bigr\Vert _{B} \geq\frac{\epsilon '-\epsilon}{3K} \biggr\} , \\ &D _{2}:= \biggl\{ n \in I_{r} : \bigl\Vert L_{n}(t, x) - x \bigr\Vert _{B} \geq\frac{\epsilon '-\epsilon}{3K} \biggr\} , \\ &D _{3}:= \biggl\{ n \in I_{r} : \bigl\Vert L_{n}\bigl(t^{2}, x\bigr) - x^{2} \bigr\Vert _{B} \geq\frac{\epsilon '-\epsilon}{3K} \biggr\} . \end{aligned}$$

Then it is easy to see that $D \subset D_{1}\cup D_{2} \cup D_{3} $. Now, from (2.13), we have

$$\begin{aligned} \bigl\vert \bigl\{ n \in I_{r} : \bigl\Vert L_{n} \bigl(g',x\bigr) - g'(x) \bigr\Vert _{B} \geq\epsilon' \bigr\} \bigr\vert \leq {}&\biggl\vert \biggl\{ n \in I_{r} : \bigl\Vert L_{n}(1, x) - 1 \bigr\Vert _{B} \geq\frac{\epsilon'-\epsilon}{3K} \biggr\} \biggr\vert \\ &{}+ \biggl\vert \biggl\{ n \in I_{r} : \bigl\Vert L_{n}(t, x) - x \bigr\Vert _{B} \geq\frac{\epsilon '-\epsilon}{3K} \biggr\} \biggr\vert \\ &{}+ \biggl\vert \biggl\{ n \in I_{r} : \bigl\Vert L_{n} \bigl(t^{2}, x\bigr) - x^{2} \bigr\Vert _{B} \geq \frac{\epsilon '-\epsilon}{3K} \biggr\} \biggr\vert , \end{aligned}$$

which yields

$$\begin{aligned} &\frac{1}{f(h_{r})} f\bigl( \bigl\vert \bigl\{ n \in I_{r} : \bigl\Vert L_{n}\bigl(g',x\bigr) - g'(x) \bigr\Vert _{B} \geq\epsilon' \bigr\} \bigr\vert \bigr) \\ &\quad \leq \frac{1}{f(h_{r})}f \biggl( \biggl\vert \biggl\{ n \in I_{r} : \bigl\Vert L_{n}(1, x) - 1 \bigr\Vert _{B} \geq \frac{\epsilon'-\epsilon}{3K} \biggr\} \biggr\vert \biggr) \\ &\qquad {}+ \frac{1}{f(h_{r})}f \biggl( \biggl\vert \biggl\{ n \in I_{r} : \bigl\Vert L_{n}(t, x) - x \bigr\Vert _{B} \geq \frac{\epsilon'-\epsilon}{3K} \biggr\} \biggr\vert \biggr) \\ &\qquad {}+ \frac{1}{f(h_{r})}f \biggl( \biggl\vert \biggl\{ n \in I_{r} : \bigl\Vert L_{n}\bigl(t^{2}, x\bigr) - x^{2} \bigr\Vert _{B} \geq\frac{\epsilon'-\epsilon}{3K} \biggr\} \biggr\vert \biggr) \end{aligned}$$

and, using (2.2)–(2.4), we get

2.14 $$ S^{f}_{\theta}-\lim \bigl\Vert L_{n} \bigl(g',x\bigr) - g'(x) \bigr\Vert _{B} = 0, \quad\text{for all } g' \in F_{cb}\bigl([a,b] \bigr). $$

Now let $g \in D([a,b])$. This implies that $g= \alpha_{1}g_{1}+\alpha _{2}g_{2}+\cdots+\alpha_{m}g_{m}$ for some $m \in\mathbb{N}$, where $\alpha_{i} \in\mathbb{R,}$ and $g_{i} \in F_{cb}([a,b])\cup\{1,t,t^{2}\}$, $1\leq i\leq m$.

Now,

$$\begin{aligned} & \bigl\Vert L_{n}(g,x) - g(x) \bigr\Vert _{B} \\ &\quad = \bigl\Vert L_{n}(\alpha_{1}g_{1}+ \alpha_{2}g_{2}+\cdots+\alpha_{m}g_{m},x) - (\alpha _{1}g_{1}+\alpha_{2}g_{2}+ \cdots+\alpha_{m}g_{m}) (x) \bigr\Vert _{B} \\ &\quad \leq K' \bigl( \bigl\Vert L_{n}(g_{1},x)-g_{1}(x) \bigr\Vert _{B} + \bigl\Vert L_{n}(g_{2},x)-g_{2}(x) \bigr\Vert _{B}+ \cdots+ \bigl\Vert L_{n}(g_{m},x)-g_{m}(x) \bigr\Vert _{B} \bigr), \end{aligned}$$

where $K'= \max\{|\alpha_{1}|, |\alpha_{2}|, \ldots, |\alpha_{m}|\}$.

Thus, for any $\epsilon''>0$, we have

$$\begin{aligned} &\frac{1}{f(h_{r})} f\bigl( \bigl\vert \bigl\{ n \in I_{r} : \bigl\Vert L_{n}(g,x) - g(x) \bigr\Vert _{B} \geq \epsilon'' \bigr\} \bigr\vert \bigr) \\ &\quad \leq \frac{1}{f(h_{r})}f \biggl( \biggl\vert \biggl\{ n \in I_{r} : \bigl\Vert L_{n}(g_{1}, x) - g_{1}(x) \bigr\Vert _{B} \geq\frac{\epsilon''}{mK'} \biggr\} \biggr\vert \biggr) \\ &\qquad {}+\frac{1}{f(h_{r})}f \biggl( \biggl\vert \biggl\{ n \in I_{r} : \bigl\Vert L_{n}(g_{2}, x) - g_{2}(x) \bigr\Vert _{B} \geq\frac{\epsilon''}{mK'} \biggr\} \biggr\vert \biggr) \\ &\qquad {}+ \cdots+ \frac{1}{f(h_{r})}f \biggl( \biggl\vert \biggl\{ n \in I_{r} : \bigl\Vert L_{n}(g_{m}, x) - g_{m}(x) \bigr\Vert _{B} \geq\frac{\epsilon''}{mK'} \biggr\} \biggr\vert \biggr) \end{aligned}$$

and, using (2.14), get

$$ S^{f}_{\theta}-\lim \bigl\Vert L_{n}(g,x) - g(x) \bigr\Vert _{B} = 0 \quad\text{for all } g \in D\bigl([a,b] \bigr). $$

 □

#### Remark 2.4

Since every convergent sequence is *f*-lacunary statistically convergent [6], it immediately follows that any sequence satisfying the conditions of the classical Korovkin first theorem automatically satisfies the conditions of its *f*-lacunary statistical analog.

Our next example shows that there may exist a sequence of positive linear operators which satisfies the conditions of Theorem 2.3 but does not satisfy the conditions of Theorem 1.12, thereby showing that our result is stronger than the classical one.

#### Example 2.5

Following Gadjiev and Orhan [18], consider the sequence $Q_{n} : D([0,1]) \to B([0,1])$ of positive linear operators defined by

$$ Q_{n}(g,x) = (1+\alpha_{n})B_{n}(g,x), $$

where $(B_{n})$ is the sequence of classical Bernstein polynomials defined by

$$ B_{n}(g,x)= \sum_{k=0}^{n}g \biggl(\frac{k}{n} \biggr)\begin{pmatrix} n\\k \end{pmatrix} x^{k}(1-x)^{n-k}; \quad0 \leq x\leq1, $$

and $(\alpha_{n})$ is the sequence of scalars which is *f*-lacunary statistically convergent to zero for some unbounded modulus *f* but not convergent to zero. Before proceeding further, we give a specific example of such type of a sequence $(\alpha_{n})$ as follows.

Let *f* be an unbounded modulus, for which $\lim_{t \to\infty}\frac {f(t)}{t} > 0$ and there is a positive constant *c* such that $f(xy)\geq cf(x)f(y)$ for all $x\geq0, y \geq0$. Proceeding as in [15, p. 511], let $\theta = (k_{r})$ be a lacunary sequence and $(k_{r(j)})$ a subsequence of lacunary sequence *θ* such that $q_{r(j)} > j$. Define a bounded sequence $\alpha=(\alpha_{k})$ by

$$\alpha_{k} = \textstyle\begin{cases} 1 &\text{if } k_{r(j)-1} < k \leq2k_{r(j)-1}, \text{ for some } j= 1,2,3,\ldots;\\ 0 & \text{otherwise}. \end{cases} $$

Then $\alpha\in N_{\theta}^{0}$ (the space of all sequences which are lacunary strongly convergent to zero). By Theorem 3 of [31], we have $N_{\theta}^{0} \subset N_{\theta,0}^{f} $ (the space of all sequences which are lacunary strongly convergent to zero with respect to *f*) and, by Theorem 14 of [6], we have $N_{\theta,0}^{f} \subset S_{\theta,0}^{f}$ (the space of all sequences which are *f*-lacunary statistically convergent to zero), from where it follows that $\alpha\in S_{\theta,0}^{f}$. Also, clearly, $\alpha\notin c$. Hence $\alpha= (\alpha_{k})$ is an example of a sequence of scalars which is *f*-lacunary statistically convergent to zero for some unbounded modulus *f* but not convergent to zero.

It is known (see [24]) that

$$ B_{n}(1,x)= 1,\qquad B_{n}(t,x)= x\quad\text{and} \quad B_{n}\bigl(t^{2},x\bigr)= x^{2}+\frac{(x-x^{2})}{n}. $$

Hence, for the sequence $(Q_{n})$ of positive linear operators, conditions (2.2)–(2.4) of Theorem 2.3 are apparently satisfied. So, we have

$$ S^{f}_{\theta}-\lim \bigl\Vert Q_{n}(g,x) - g(x) \bigr\Vert _{B} = 0 \quad\text{for all } g \in D\bigl([0,1] \bigr). $$

On the other hand,

$$ Q_{n}(1,x) = (1+\alpha_{n})B_{n}(1,x) = (1+ \alpha_{n}), $$

and so

$$\begin{aligned} \lim \bigl\Vert Q_{n}(1,x) - 1 \bigr\Vert _{B} = \lim \Vert 1+\alpha_{n} -1 \Vert = \lim \Vert \alpha_{n} \Vert =\lim \vert \alpha_{n} \vert \neq0, \end{aligned}$$

from where it follows that $(Q_{n})$ does not satisfy the conditions of classical Korovkin first theorem.

#### Remark 2.6

Since every *f*-lacunary statistically convergent sequence is lacunary statistically convergent [6], it immediately follows that any sequence satisfying the conditions of the *f*-lacunary statistical analog of the classical Korovkin first theorem (Theorem 2.3) automatically satisfies the conditions of the lacunary statistical analog of the classical Korovkin first theorem (Theorem 1.18).

We next claim that the lacunary statistical analog of the classical Korovkin first theorem is stronger than the *f*-lacunary statistical analog of the classical Korovkin first theorem. For this we first provide an example of a lacunary statistically convergent sequence which is not *f*-lacunary statistically convergent.

#### Example 2.7

Consider the lacunary sequence $\theta=(k_{r})$ defined as follows:

$$\begin{aligned} &k_{0}=0, \\ &k_{r}=r+k_{r-1}, \quad r=1,2,\ldots. \end{aligned}$$

Then $h_{r}=r, r=1,2,\ldots$ . Now we define a sequence $x=(x_{k})$ of scalars by defining it on the intervals $I_{r}=(k_{r-1},k_{r}], r=1,2,\dots $ determined by *θ*. Set $x_{0}=0$ and let

$$\begin{aligned} &x_{k_{r}}=x_{k_{r}-1}=\cdots=x_{k_{r}-[\sqrt{r}]}=1, \\ &x_{k_{r}-[\sqrt{r}]-1}=\cdots=x_{k_{r-1}+1}=0, \end{aligned}$$

where $[\sqrt{r}]$ denotes the greatest integer less than or equal to $\sqrt{r}$.

*Aside.* For a better understanding let us write down first a few terms of *θ* and *x*:

$$\begin{aligned} &\theta=(0,1,3,6,10,15,21,28,36,45,55,66,78,91,105,\dots), \\ &x=(\underbrace{0}_{x_{0}},\underbrace{1}_{I_{1}},\underbrace {1,1}_{I_{2}},\underbrace{0,1,1}_{I_{3}},\underbrace {0,1,1,1}_{I_{4}},\underbrace{0,0,1,1,1}_{I_{5}},\underbrace {0,0,0,1,1,1}_{I_{6}},\underbrace{0,0,0,0,1,1,1}_{I_{7}},\ldots). \end{aligned}$$

Now for any $\varepsilon>1$,

$$\bigl\vert \bigl\{ k\in I_{r}: \vert x_{k} \vert \geq \varepsilon\bigr\} \bigr\vert =0\quad \Longrightarrow\quad \lim_{r\to\infty } \frac{ \vert \{k\in I_{r}: \vert x_{k} \vert \geq\varepsilon\} \vert }{h_{r}}=0. $$

For $0<\varepsilon\leq1$, by construction of the sequence $x=(x_{k})$, we have

$$\bigl\vert \bigl\{ k\in I_{r}: \vert x_{k} \vert \geq \varepsilon\bigr\} \bigr\vert =[\sqrt{r}]\quad \Longrightarrow\quad \lim _{r\to \infty}\frac{ \vert \{k\in I_{r}: \vert x_{k} \vert \geq\varepsilon\} \vert }{h_{r}}=\lim_{r\to\infty } \frac{[\sqrt{r}]}{r}=0 $$

because

$$\frac{\sqrt{r}-1}{r}\leq\frac{[\sqrt{r}]}{r}\leq\frac{\sqrt{r}}{r}. $$

Therefore,

$$S_{\theta}-\lim x_{k}=0. $$

Now consider the unbounded modulus function $f(x)=\log(1+x)$. We will show that $S^{f}_{\theta}-\lim x_{k}\neq0$, whence it will follow that $(x_{k})$ is not *f*-lacunary statistically convergent. Indeed, suppose $(x_{k})$ were *f*-lacunary statistically convergent to some number *l*, then by Theorem 11 of [6], $(x_{k})$ would be lacunary statically convergent to *l* and, finally, by the uniqueness of $S_{\theta}$-limit for a fixed *θ* (see [17], page 48), this *l* had to be 0.

It is clear that for $\varepsilon>1$, $f(|\{k\in I_{r}:|x_{k}|\geq \varepsilon\}|)=f(0)=0$, and hence

$$\lim_{r\to\infty}\frac{f( \vert \{k\in I_{r}: \vert x_{k} \vert \geq\varepsilon\} \vert )}{f(h_{r})}=0. $$

Now note that

$$\frac{\log\sqrt{r}}{\log(r+1)}\leq\frac{\log([\sqrt{r}]+1)}{\log (r+1)}\leq\frac{\log(\sqrt{r}+1)}{\log(r+1)}, $$

and so

$$\lim_{r\to\infty}\frac{\log\sqrt{r}}{\log(r+1)}=\frac{1}{2}=\lim _{r\to \infty}\frac{\log(\sqrt{r}+1)}{\log(r+1)}. $$

Therefore, for $0<\varepsilon\leq1$,

$$\lim_{r\to\infty}\frac{f( \vert \{k\in I_{r}: \vert x_{k} \vert \geq\varepsilon\} \vert )}{f(h_{r})}=\lim_{r\to\infty} \frac{f([\sqrt{r}])}{f(r)}=\lim_{r\to\infty }\frac{\log([\sqrt{r}]+1)}{\log(r+1)}= \frac{1}{2}. $$

Therefore, $x=(x_{k})$ is not *f*-lacunary statistically convergent, and hence the inclusion $S^{f}_{\theta}\subset S_{\theta}$ may be strict, in general.

Our next example shows that there exists a sequence of positive linear operators which satisfies the conditions of Theorem 1.18 but does not satisfy the conditions of Theorem 2.3, thereby implying that the lacunary statistical analog of the classical Korovkin first theorem is stronger than the *f*-lacunary statistical analog of the classical Korovkin first theorem.

#### Example 2.8

Consider the sequence $Q_{n} : D([0,1]) \to B([0,1])$ of positive linear operators defined by

$$ Q_{n}(g,x) = (1+\alpha_{n})B_{n}(g,x), $$

where $(B_{n})$ is the sequence of classical Bernstein polynomials and $(\alpha_{n})$ is any sequence of scalars which is lacunary statistically convergent to zero but not *f*-lacunary statistically convergent to zero for some unbounded modulus *f*. It is easy to see, as in Example 2.5, that the sequence $(Q_{n})$ satisfies the lacunary statistical analog of Korovkin first theorem but does not satisfy the *f*-lacunary statistical analog of Korovkin first theorem.

We now study a relationship between the *f*-lacunary statistical analog and the *f*-statistical analog of the Korovkin first theorem. In other words, we characterize those *θ* for which these two analogs become equivalent, of course, under certain restrictions on *f*. In order to do this, we need the following lemmas which are actually simple extensions of Lemmas 17 and 19 of Bhardwaj and Dhawan [6] to an arbitrary normed space.

#### Lemma 2.9

*In a normed space* *X*, *for any lacunary sequence* *θ* *and unbounded modulus f*, *for which* $\lim_{t \to\infty}\frac{f(t)}{t} > 0$ *and there is a positive constant* *c* *such that* $f(xy)\geq cf(x)f(y)$ *for all* $x\geq0, y \geq0$, *one has* $S^{f}(X) \subset S_{\theta}^{f}(X)$ *if and only if* $\liminf_{r} q_{r} >1$.

#### Lemma 2.10

*In a normed space* *X*, *for any lacunary sequence* *θ* *and unbounded modulus f*, *for which* $\lim_{t \to\infty}\frac{f(t)}{t} > 0$ *and there is a positive constant* *c* *such that* $f(xy)\geq cf(x)f(y)$ *for all* $x\geq0, y \geq0$, *one has* $S_{\theta}^{f}(X) \subset S^{f}(X)$ *if and only if* $\limsup_{r} q_{r} < \infty$.

Combining Lemmas 2.9 and 2.10, we have the following.

#### Theorem 2.11

*In a normed space* *X*, *for any lacunary sequence* *θ* *and unbounded modulus f*, *for which* $\lim_{t \to\infty}\frac{f(t)}{t} > 0$ *and there is a positive constant* *c* *such that* $f(xy)\geq cf(x)f(y)$ *for all* $x\geq0, y \geq0$, *one has* $S_{\theta}^{f}(X) = S^{f}(X)$ *if and only if* $1 <\liminf_{r} q_{r} \leq\limsup_{r} q_{r} < \infty$.

In view of Theorems 1.19, 2.3 and 2.11, we immediately have the following.

#### Theorem 2.12

*Let* *f* *be any unbounded modulus*, *for which* $\lim_{t \to\infty}\frac{f(t)}{t} > 0$ *and there is a positive constant* *c* *such that* $f(xy)\geq cf(x)f(y)$ *for all* $x\geq0, y \geq0$. *Then*, *the* *f*-*lacunary statistical analog and* *f*-*statistical analog of the Korovkin first theorem are equivalent for those* *θ* *for which* $1 <\liminf_{r} q_{r} \leq\limsup_{r} q_{r} < \infty$.

### *f*-lacunary statistical analog of Korovkin second theorem

The classical Korovkin second theorem [24] may be stated as follows.

#### Theorem 2.13

*If the sequence* $(L_{n})$ *of positive linear operators* $L_{n} : D^{*}([a,b]) \to B([a,b])$ *satisfies the conditions*

$$\begin{aligned} &\lim \bigl\Vert L_{n}(1,x) - 1 \bigr\Vert _{B} = 0, \\ &\lim \bigl\Vert L_{n}(\cos t,x) - \cos x \bigr\Vert _{B} = 0, \\ &\lim \bigl\Vert L_{n}(\sin t,x) - \sin x \bigr\Vert _{B} = 0, \end{aligned}$$

*then for any function* $g \in F_{cb}^{*}([a,b])$, *we have*

$$ \lim \bigl\Vert L_{n}(g,x) - g(x) \bigr\Vert _{B} = 0. $$

We now prove an *f*-lacunary statistical analog of the Korovkin second theorem, from which the lacunary statistical analog is obtained as a particular case.

#### Theorem 2.14

*Let* *f* *be an unbounded modulus and* $(L_{n})$ *be a sequence of positive linear operators* $L_{n} : D^{*}([a,b]) \to B([a,b])$. *Then*, *for all* $g \in D^{*}([a,b])$,

2.15 $$ S^{f}_{\theta}-\lim \bigl\Vert L_{n}(g,x) - g(x) \bigr\Vert _{B} = 0 $$

*if and only if*

2.16 $$\begin{aligned} &S^{f}_{\theta}-\lim \bigl\Vert L_{n}(1,x) - 1 \bigr\Vert _{B} = 0, \end{aligned}$$

2.17 $$\begin{aligned} &S^{f}_{\theta}-\lim \bigl\Vert L_{n}(\cos t,x) - \cos x \bigr\Vert _{B} = 0, \end{aligned}$$

2.18 $$\begin{aligned} &S^{f}_{\theta}-\lim \bigl\Vert L_{n}(\sin t,x) - \sin x \bigr\Vert _{B} = 0 . \end{aligned}$$

#### Proof

Since each of $1, \cos t, \sin t$ belongs to $D^{*}([a,b])$, conditions (2.16)–(2.18) follow immediately from (2.15). Now, let the conditions (2.16)–(2.18) hold. To prove (2.15), we first prove that for any $g' \in F_{cb}^{*}([a,b])$,

$$ S^{f}_{\theta}-\lim \bigl\Vert L_{n} \bigl(g',x\bigr) - g'(x) \bigr\Vert _{B} = 0. $$

We follow the proof of Theorem 2 of Korovkin [24] up to a certain stage. Since the function $g'$ is bounded on the whole real axis, we can write

2.19 $$ \bigl\vert g'(t) - g'(x) \bigr\vert < 2M, \quad-\infty< t, x < \infty. $$

Further, in view of Lemma 2.1, there exists a $\delta> 0$ for each $\epsilon> 0$ such that

2.20 $$ \bigl\vert g'(t) - g'(x) \bigr\vert < \epsilon\quad\text{for } a\leq x \leq b, \vert t-x \vert < \delta. $$

Now we take the subinterval $x-\delta< t \leq2\pi+ x-\delta$ of length 2*π*, where $x \in[a,b]$ is fixed. Taking $\psi(t) = \sin^{2}\frac{(t-x)}{2}$ and using (2.19) and (2.20), we have

$$ \bigl\vert g'(t)-g'(x) \bigr\vert < \epsilon+ \frac{2M}{\sin^{2}\frac{\delta}{2}}\psi(t) \quad\text{for all } t \in(x-\delta, 2\pi+ x - \delta]. $$

This means

2.21 $$\begin{aligned} &{-}\epsilon- \frac{2M}{\sin^{2}\frac{\delta}{2}}\psi(t) < g'(t) - g'(x) < \epsilon+ \frac{2M}{\sin^{2}\frac{\delta}{2}}\psi(t) \\ &\quad\text{for all } t \in(x-\delta, 2\pi+ x - \delta]. \end{aligned}$$

In fact, if $|t-x|<\delta$, then inequality (2.21) follows from (2.20), since $\psi(t) = \sin^{2}\frac{(t-x)}{2} \geq0$. If $\delta\leq t-x \leq2\pi-\delta$, then $\frac{\delta}{2} \leq\frac {t-x}{2} \leq\pi- \frac{\delta}{2}$, and thus $\sin(\frac{t-x}{2}) \geq\sin \frac{\delta}{2} $, $\psi(t) = \sin^{2} (\frac{t-x}{2}) \geq \sin^{2} \frac{\delta}{2}$, $\frac{2M}{\sin^{2}\frac{\delta}{2}}\psi(t) \geq2M$, and inequality (2.21) follows from inequality (2.19) since $\epsilon>0$.

Thus inequality (2.21), on which the proof of this theorem is based, has not yet been established for all *t*, but it holds for *t* belonging to the subinterval $(x-\delta, 2\pi+ x - \delta]$ of length 2*π*. In order to prove validity of inequality (2.21) for all $t \in(-\infty, \infty)$, we note that the function

$$ \psi(t) = \sin^{2} \biggl(\frac{t-x}{2} \biggr) = \frac{1-\cos(t-x)}{2} $$

has period 2*π* and, according to the conditions of the theorem, the function $g'(t)$ also has period 2*π*, i.e.,

$$ \psi(t+2k\pi) = \psi(t) \quad\text{and}\quad g'(t+2k\pi)= g'(t). $$

Therefore, we find from (2.21) that

2.22 $$ -\epsilon- \frac{2M}{\sin^{2}\frac{\delta}{2}}\psi(t+2k\pi) < g'(t+2k \pi ) - g'(x) < \epsilon+ \frac{2M}{\sin^{2}\frac{\delta}{2}}\psi(t+2k\pi). $$

But if *t* varies in the subinterval $(x-\delta, 2\pi+ x - \delta]$, then $t+2\pi$ will vary in the subinterval $(2\pi+x-\delta, 4\pi+ x - \delta]$, $t+4\pi$ in the subinterval $(4\pi+x-\delta, 6\pi+ x - \delta]$, and, in general, $t+2k\pi$ will vary in the subinterval $(2k\pi+x-\delta, 2k\pi+2\pi+ x - \delta]$, $k = 0,\pm1, \pm 2, \pm3, \ldots$ .

The totality of these subintervals covers without any gap the whole real axis, and thus the inequality (2.21), whose validity on every subinterval follows from (2.22), is proved for all values of *t*.

Using inequality (2.21) and monotonicity of the operator $L_{n}(g',x)$, we obtain

$$ -\epsilon L_{n}(1,x) - \frac{2M}{\sin^{2}\frac{\delta}{2}}L_{n}(\psi,x) \leq L_{n}\bigl(g',x\bigr) - L_{n} \bigl(g'(x), x\bigr) \leq\epsilon L_{n}(1,x) + \frac{2M}{\sin^{2}\frac {\delta}{2}}L_{n}(\psi,x). $$

As we know *x* is fixed and so $g'(x)$ is a constant number. Therefore,

2.23 $$\begin{aligned} &{-} \epsilon L_{n}(1,x) - \frac{2M}{\sin^{2}\frac{\delta}{2}}L_{n}( \psi,x) \\ &\quad \leq L_{n}\bigl(g',x\bigr)-g'(x) L_{n}(1,x) \leq \epsilon L_{n}(1,x) + \frac{2M}{\sin ^{2}\frac{\delta}{2}}L_{n}( \psi,x) . \end{aligned}$$

Also,

2.24 $$ L_{n}\bigl(g',x\bigr) - g'(x) = \bigl[L_{n}\bigl(g',x\bigr) - g'(x)L_{n}(1, x) \bigr] + g'(x) \bigl[L_{n}(1, x) - 1\bigr]. $$

From (2.23) and (2.24), we have

2.25 $$ L_{n}\bigl(g',x\bigr) - g'(x) \leq\epsilon L_{n}(1,x) + \frac{2M}{\sin^{2}\frac{\delta }{2}}L_{n}(\psi,x) + g'(x) \bigl[L_{n}(1, x) - 1\bigr]. $$

Now,

$$\begin{aligned} L_{n}(\psi,x) &= L_{n}\biggl(\sin^{2} \frac{(t-x)}{2}, x\biggr) \\ &= L_{n} \biggl(\frac{1}{2}(1-\cos t \cos x - \sin t \sin x),x \biggr) \\ &=\frac{1}{2} \bigl\{ L_{n}(1,x) - \cos xL_{n}(\cos t,x) - \sin xL_{n}(\sin t,x) \bigr\} \\ &= \frac{1}{2} \bigl\{ \bigl[L_{n}(1, x) - 1\bigr]- \cos x \bigl[L_{n}(\cos t, x) - \cos x\bigr]- \sin x\bigl[L_{n}(\sin t, x) - \sin x\bigr] \bigr\} . \end{aligned}$$

Substituting the value of $L_{n}(\psi,x)$ in (2.25), we get

$$\begin{aligned} &L_{n}\bigl(g',x\bigr) - g'(x) \\ &\quad \leq\epsilon L_{n}(1,x) \\ &\qquad {}+ \frac{M}{\sin^{2}\frac{\delta}{2}} \bigl\{ \bigl[L_{n}(1, x) - 1\bigr]- \cos x\bigl[L_{n}(\cos t, x) - \cos x\bigr]-\sin x\bigl[L_{n}(\sin t, x) - \sin x\bigr] \bigr\} \\ &\qquad {} + g'(x) \bigl[L_{n}(1, x) - 1\bigr] \\ &\quad =\frac{M}{\sin^{2}\frac{\delta}{2}} \bigl\{ \bigl[L_{n}(1, x) - 1\bigr]- \cos x \bigl[L_{n}(\cos t, x) - \cos x\bigr]- \sin x\bigl[L_{n}(\sin t, x) - \sin x\bigr] \bigr\} \\ &\qquad {} + \epsilon\bigl[L_{n}(1,x) - 1\bigr] + \epsilon+ g'(x) \bigl[L_{n}(1, x) - 1\bigr] \\ &\quad = \biggl(\epsilon+\frac{M}{\sin^{2}\frac{\delta}{2}}+g'(x) \biggr) \bigl[L_{n}(1, x) - 1\bigr]- \frac{M}{\sin^{2}\frac{\delta}{2}}\cos x \bigl[L_{n}(\cos t, x) - \cos x\bigr] \\ &\qquad {} -\frac{M}{\sin^{2}\frac{\delta}{2}}\sin x\bigl[L_{n}(\sin t, x) - \sin x\bigr]+ \epsilon. \end{aligned}$$

Since $|g'(x)|\leq M$, $|\cos x|\leq1$ and $|\sin x|\leq1$, for all $x \in[a,b]$,

2.26 $$\begin{aligned} & \bigl\Vert L_{n}\bigl(g',x\bigr) - g'(x) \bigr\Vert _{B} \\ &\quad \leq \biggl(\epsilon+\frac{M}{\sin^{2}\frac{\delta}{2}}+M \biggr) \bigl\Vert L_{n}(1, x) - 1 \bigr\Vert _{B} + \frac{M}{\sin^{2}\frac{\delta}{2}} \bigl\Vert L_{n}(\cos t, x) - \cos x \bigr\Vert _{B} \\ &\qquad {}+ \frac{M}{\sin^{2}\frac{\delta}{2}} \bigl\Vert L_{n}(\sin t, x) - \sin x \bigr\Vert _{B} + \epsilon \\ &\quad \leq K \bigl( \bigl\Vert L_{n}(1, x) - 1 \bigr\Vert _{B}+ \bigl\Vert L_{n}(\cos t, x) - \cos x \bigr\Vert _{B}+ \bigl\Vert L_{n}(\sin t, x) - \sin x \bigr\Vert _{B} \bigr) +\epsilon, \end{aligned}$$

where $K = \max \{\epsilon+\frac{M}{\sin^{2}\frac{\delta}{2}}+M, \frac {M}{\sin^{2}\frac{\delta}{2}} \}$.

For any $\epsilon'>0$, choose $\epsilon>0$ such that $\epsilon<\epsilon '$. Now, from inequality (2.26), we have

2.27 $$\begin{aligned} & \bigl\vert \bigl\{ n \in I_{r} : \bigl\Vert L_{n} \bigl(g',x\bigr) - g'(x) \bigr\Vert _{B} \geq\epsilon' \bigr\} \bigr\vert \\ &\quad \leq \biggl\vert \biggl\{ n \in I_{r} : \bigl\Vert L_{n}(1, x) - 1 \bigr\Vert _{B}+ \bigl\Vert L_{n}(\cos t, x) - \cos x \bigr\Vert _{B} \\ &\qquad {}+ \bigl\Vert L_{n}(\sin t, x) - \sin x \bigr\Vert _{B} \geq \frac{\epsilon'-\epsilon }{K} \biggr\} \biggr\vert . \end{aligned}$$

Now write

$$\begin{aligned} &D := \biggl\{ n : \bigl\Vert L_{n}(1, x) - 1 \bigr\Vert _{B}+ \bigl\Vert L_{n}(\cos t, x) - \cos x \bigr\Vert _{B}+ \bigl\Vert L_{n}(\sin t, x) - \sin x \bigr\Vert _{B} \geq\frac{\epsilon'-\epsilon}{K} \biggr\} , \\ &D _{1} := \biggl\{ n : \bigl\Vert L_{n}(1, x) - 1 \bigr\Vert _{B} \geq\frac{\epsilon'-\epsilon }{3K} \biggr\} , \\ &D _{2} := \biggl\{ n : \bigl\Vert L_{n}(\cos t, x) -\cos x \bigr\Vert _{B} \geq\frac{\epsilon '-\epsilon}{3K} \biggr\} , \\ &D _{3} := \biggl\{ n : \bigl\Vert L_{n}(\sin t, x) - \sin x \bigr\Vert _{B} \geq\frac{\epsilon '-\epsilon}{3K} \biggr\} . \end{aligned}$$

Then it is easy to see that $D \subset D_{1}\cup D_{2} \cup D_{3} $. Now, from (2.27), we have

$$\begin{aligned} \bigl\vert \bigl\{ n \in I_{r} : \bigl\Vert L_{n} \bigl(g',x\bigr) - g'(x) \bigr\Vert _{B} \geq\epsilon' \bigr\} \bigr\vert \leq{} &\biggl\vert \biggl\{ n\in I_{r} : \bigl\Vert L_{n}(1, x) - 1 \bigr\Vert _{B} \geq\frac{\epsilon'-\epsilon}{3K} \biggr\} \biggr\vert \\ &{}+ \biggl\vert \biggl\{ n \in I_{r} : \bigl\Vert L_{n}( \cos t, x) - \cos x \bigr\Vert _{B} \geq\frac {\epsilon'-\epsilon}{3K} \biggr\} \biggr\vert \\ &{}+ \biggl\vert \biggl\{ n \in I_{r}: \bigl\Vert L_{n}( \sin t, x) - \sin x \bigr\Vert _{B} \geq\frac {\epsilon'-\epsilon}{3K} \biggr\} \biggr\vert , \end{aligned}$$

which yields

$$\begin{aligned} &\frac{1}{f(h_{r})} f\bigl( \bigl\vert \bigl\{ n \in I_{r} : \bigl\Vert L_{n}\bigl(g',x\bigr) - g'(x) \bigr\Vert _{B} \geq\epsilon' \bigr\} \bigr\vert \bigr) \\ &\quad \leq \frac{1}{f(h_{r})}f \biggl( \biggl\vert \biggl\{ n \in I_{r} : \bigl\Vert L_{n}(1, x) - 1 \bigr\Vert _{B} \geq \frac{\epsilon'-\epsilon}{3K} \biggr\} \biggr\vert \biggr) \\ &\qquad {}+ \frac{1}{f(h_{r})}f \biggl( \biggl\vert \biggl\{ n\in I_{r} : \bigl\Vert L_{n}(\cos t, x) - \cos x \bigr\Vert _{B} \geq \frac{\epsilon'-\epsilon}{3K} \biggr\} \biggr\vert \biggr) \\ &\qquad {}+ \frac{1}{f(h_{r})}f \biggl( \biggl\vert \biggl\{ n \in I_{r} : \bigl\Vert L_{n}(\sin t, x) - \sin x \bigr\Vert _{B} \geq \frac{\epsilon'-\epsilon}{3K} \biggr\} \biggr\vert \biggr) \end{aligned}$$

and, using (2.16)–(2.18)), we get

$$ S^{f}_{\theta}-\lim \bigl\Vert L_{n} \bigl(g',x\bigr) - g'(x) \bigr\Vert _{B} = 0 \quad\text{for all } g' \in F_{cb}^{*}\bigl([a,b] \bigr). $$

From here onwards, we proceed as in the proof of Theorem 2.3 to get

$$ S^{f}_{\theta}-\lim \bigl\Vert L_{n}(g,x) - g(x) \bigr\Vert _{B} = 0 \quad\text{for all } g \in D^{*}\bigl([a,b] \bigr). $$

 □

#### Remark 2.15

If we take $f(x) = x$ in Theorem 2.14, we obtain the lacunary statistical analog of the classical Korovkin second theorem as follows.

#### Theorem 2.16

*Let* $(L_{n})$ *be a sequence of positive linear operators* $L_{n} : D^{*}([a,b]) \to B([a,b])$. *Then*, *for all* $g \in D^{*}([a,b])$,

$$ S_{\theta}-\lim \bigl\Vert L_{n}(g,x) - g(x) \bigr\Vert _{B} = 0 $$

*if and only if*

$$\begin{aligned} &S_{\theta}-\lim \bigl\Vert L_{n}(1,x) - 1 \bigr\Vert _{B} = 0, \\ &S_{\theta}-\lim \bigl\Vert L_{n}(\cos t,x) - \cos x \bigr\Vert _{B} = 0, \\ &S_{\theta}-\lim \bigl\Vert L_{n}(\sin t,x) - \sin x \bigr\Vert _{B} = 0. \end{aligned}$$

#### Remark 2.17

Since every convergent sequence is *f*-lacunary statistically convergent [6], it immediately follows that any sequence satisfying the conditions of the classical Korovkin second theorem automatically satisfies the conditions of its *f*-lacunary statistical analog.

Our next example shows that there exists a sequence of positive linear operators which satisfies the conditions of Theorem 2.14 but does not satisfy the conditions of Theorem 2.13, thereby showing that our result is stronger than the classical one.

#### Example 2.18

Following Duman [13], consider the sequence $Q_{n} : D^{*}([-\pi,\pi ]) \to B([-\pi,\pi])$ of positive linear operators defined by

$$ Q_{n}(g,x) = (1+\alpha_{n})F_{n}(g,x), $$

where $(F_{n})$ is the sequence of Fejer operators defined by

$$ F_{n}(g,x)= \frac{1}{n\pi} \int_{-\pi}^{\pi}g(t)\frac{\sin^{2} (\frac {n}{2}(t-x ))}{2 \sin^{2} (\frac{t-x}{2} )}\,dt, \quad g \in D^{*}\bigl([-\pi,\pi]\bigr), $$

and $(\alpha_{n})$ is the sequence of scalars which is *f*-lacunary statistically convergent to zero for some unbounded modulus *f* but not convergent to zero. It is known [24] that

$$ F_{n}(1,x)= 1,\qquad F_{n}(\cos t,x)= \frac{n-1}{n}\cos x\quad\text{and} \quad F_{n}(\sin t ,x)= \frac{n-1}{n}\sin x. $$

Hence, the sequence $(Q_{n})$ satisfies conditions (2.16)–(2.18) of Theorem 2.14. So, we have

$$ S^{f}_{\theta}-\lim \bigl\Vert Q_{n}(g,x) - g(x) \bigr\Vert _{B} = 0 \quad\text{for all } g \in D^{*}\bigl([-\pi, \pi]\bigr). $$

On the other hand,

$$ Q_{n}(1,x) = (1+\alpha_{n})F_{n}(1,x) = (1+ \alpha_{n}), $$

and so,

$$\begin{aligned} \lim \bigl\Vert Q_{n}(1,x) - 1 \bigr\Vert _{B} = \lim \Vert 1+\alpha_{n} -1 \Vert = \lim \Vert \alpha_{n} \Vert =\lim \vert \alpha_{n} \vert \neq0, \end{aligned}$$

from where it follows that $(Q_{n})$ does not satisfy the conditions of classical Korovkin second theorem.

#### Remark 2.19

Since every *f*-lacunary statistically convergent sequence is lacunary statistically convergent [6], it immediately follows that any sequence satisfying the conditions of the *f*-lacunary statistical analog of the classical Korovkin second theorem (Theorem 2.14) automatically satisfies the conditions of the lacunary statistical analog of the classical Korovkin second theorem (Theorem 2.16).

#### Remark 2.20

In Example 2.18, if we take $(\alpha_{n})$ to be any sequence which is lacunary statistically convergent to zero but not *f*-lacunary statistically convergent to zero for some unbounded modulus *f*, then we obtain a sequence of positive linear operators which satisfies the conditions of Theorem 2.16 but does not satisfy the conditions of Theorem 2.14, thereby showing that the lacunary statistical analog of the classical Korovkin second theorem is stronger than the *f*-lacunary statistical analog of the classical Korovkin second theorem.

We conclude this section by studying a relationship between the *f*-lacunary statistical analog and the *f*-statistical analog of the Korovkin second theorem. In other words, we characterize those *θ* for which these two analogs are equivalent, of course, under certain restrictions on *f*. In view of Theorems 1.20, 2.11 and 2.14, we have the following.

#### Theorem 2.21

*Let* *f* *be any unbounded modulus*, *for which* $\lim_{t \to\infty}\frac{f(t)}{t} > 0$ *and there is a positive constant* *c* *such that* $f(xy)\geq cf(x)f(y)$ *for all* $x\geq0, y \geq0$. *Then*, *the* *f*-*lacunary statistical analog and* *f*-*statistical analog of the Korovkin second theorem are equivalent for those* *θ* *for which* $1 <\liminf_{r} q_{r} \leq\limsup_{r} q_{r} < \infty$.

### The order of *f*-lacunary statistical convergence

The idea of lacunary statistical convergence with degree *β* $(0<\beta<1)$ for sequences of numbers was introduced by Patterson and Savaş [30] as follows:

#### Definition 2.22

The number sequence $(x_{k})$ is said to be lacunary statistically convergent to the number *l* with degree $0 < \beta<1$ if, for each $\epsilon>0$,

$$ \lim_{r \to\infty}\frac{1}{h_{r}^{1-\beta}} \bigl\vert \bigl\{ k \in I_{r} : \vert x_{k} - l \vert \geq\epsilon\bigr\} \bigr\vert =0. $$

In this case, we write

$$ x_{k} - l = S_{\theta}-o\bigl(k^{-\beta}\bigr). $$

The concept of lacunary statistical convergence of order *α* was introduced by Şengül and Et [36] as follows:

#### Definition 2.23

Let $0<\alpha\leq1$. The number sequence $(x_{k})$ is said to be lacunary statistically convergent of order *α* to the number *l* if, for each $\epsilon>0$,

$$ \lim_{r \to\infty}\frac{1}{h_{r}^{\alpha}} \bigl\vert \bigl\{ k \in I_{r} : \vert x_{k} - l \vert \geq\epsilon\bigr\} \bigr\vert =0. $$

From Definitions 2.22 and 2.23, we have the following

#### Remark 2.24

A sequence is lacunary statistically convergent of degree *β* if and only if it is lacunary statistically convergent of order $1-\beta$, where $0<\beta<1$.

We now introduce a new concept of *f*-lacunary statistical convergence with degree *β* $(0<\beta<1)$ for *X*-valued sequences, where *X* is a normed space.

#### Definition 2.25

Let *X* be a normed space. A sequence $(x_{k})$ in *X* is said to be *f*-lacunary statistically convergent to some $x \in X$ with degree $0 < \beta<1$ if, for each $\epsilon>0$,

$$ \lim_{r \to\infty}\frac{1}{f(h_{r}^{1-\beta})}f\bigl( \bigl\vert \bigl\{ k \in I_{r} : \Vert x_{k} - x \Vert \geq\epsilon\bigr\} \bigr\vert \bigr)=0. $$

In this case, we write

$$ x_{k} - x = S^{f}_{\theta}-o\bigl(k^{-\beta} \bigr). $$

#### Remark 2.26

In case $f(x) = x$, the concept of *f*-lacunary statistical convergence with degree *β* $(0<\beta<1)$ reduces to that of lacunary statistical convergence with degree *β* $(0<\beta<1)$.

#### Theorem 2.27

*Let* *f* *be an unbounded modulus and* $\theta= (k_{r})$ *be a lacunary sequence*. *Let* $(x_{k})$ *and* $(y_{k})$ *be any two sequences such that* $x_{k} - x = S^{f}_{\theta}-o(k^{-\beta_{1}})$ *and* $y_{k} - y = S^{f}_{\theta }-o(k^{-\beta_{2}})$. *Then*

(i) $(x_{k} +y_{k}) - (x+y) = S^{f}_{\theta}-o(k^{-\beta})$, *where* $\beta= \min{(\beta_{1}, \beta_{2})}$,
(ii) $\alpha(x_{k} - x) =S^{f}_{\theta}-o(k^{-\beta_{1}}) $ *for any real number* *α*.

The proof is a routine verification by using standard techniques and hence omitted.

#### Theorem 2.28

*Every* *f*-*lacunary statistically convergent sequence with degree* $0<\beta<1$ *is* *f*-*lacunary statistically convergent for any unbounded modulus* *f*.

#### Proof

Since $0<\beta<1$, we get $0<1-\beta<1$. This implies that $f(h_{r}^{1-\beta}) \leq f(h_{r})$ and hence,

$$ \frac{1}{f(h_{r})}f\bigl( \bigl\vert \bigl\{ k \in I_{r} : \Vert x_{k} - x \Vert \geq\epsilon\bigr\} \bigr\vert \bigr) \leq \frac{1}{f(h_{r}^{1-\beta})}f\bigl( \bigl\vert \bigl\{ k \in I_{r} : \Vert x_{k} - x \Vert \geq\epsilon\bigr\} \bigr\vert \bigr). $$

 □

#### Theorem 2.29

*Let* $0 < \alpha\leq\beta<1$. *If a sequence is* *f*-*lacunary statistically convergent with degree* *β* *then it is lacunary statistically convergent with degree* *α*.

#### Proof

Suppose $(x_{k})$ is *f*-lacunary statistically convergent with degree *β* to *l*. Then,

$$ \lim_{r \to\infty}\frac{1}{f(h_{r}^{1-\beta})}f\bigl( \bigl\vert \bigl\{ k \in I_{r} : \Vert x_{k} - x \Vert \geq\epsilon\bigr\} \bigr\vert \bigr)=0. $$

This implies that for a given $p \in\mathbb{N}$, there exists an $n_{o} \in\mathbb{N}$ such that for $n \geq n_{o}$, we have

$$ f \bigl( \bigl\vert \bigl\{ k \in I_{r} : \Vert x_{k} - x \Vert \geq\epsilon\bigr\} \bigr\vert \bigr) \leq\frac{1}{p}f \bigl(h_{r}^{1-\beta}\bigr) \leq \frac{1}{p}f \bigl(h_{r}^{1-\alpha}\bigr) \leq \frac{1}{p} pf \biggl( \frac{h_{r}^{1-\alpha}}{p} \biggr) = f \biggl(\frac{h_{r}^{1-\alpha}}{p} \biggr) $$

and, since *f* is increasing, we have

$$ \frac{1}{h_{r}^{1-\alpha}} \bigl\vert \bigl\{ k \in I_{r} : \Vert x_{k} - x \Vert \geq\epsilon\bigr\} \bigr\vert \leq \frac{1}{p}. $$

Hence, $(x_{k})$ is lacunary statistically convergent with degree *α*. □

Maddox [26] proved that for any modulus *f*, $\lim_{t \to\infty }\frac{f(t)}{t}$ exists. Making use of this result, we are now in a position to find the degree of *f*-lacunary statistical convergence of the sequence of positive linear operators in Theorem 2.3.

#### Theorem 2.30

*Let* *f* *be an unbounded modulus such that* $\lim_{t \to\infty}\frac {f(t)}{t} >0$. *Let* $(L_{n})$ *be a sequence of positive linear operators* $L_{n} : D([a,b]) \to B([a,b])$ *satisfy the conditions*

2.28 $$\begin{aligned} &\bigl\Vert L_{n}(1,x) - 1 \bigr\Vert _{B} = S^{f}_{\theta}-o\bigl(n^{-\beta_{1}}\bigr), \end{aligned}$$

2.29 $$\begin{aligned} &\bigl\Vert L_{n}(t,x) - x \bigr\Vert _{B} = S^{f}_{\theta}-o\bigl(n^{-\beta_{2}}\bigr), \end{aligned}$$

2.30 $$\begin{aligned} &\bigl\Vert L_{n}\bigl(t^{2},x\bigr) - x^{2} \bigr\Vert _{B} = S^{f}_{\theta}-o \bigl(n^{-\beta_{3}}\bigr) . \end{aligned}$$

*Then*, *for all* $g' \in F_{cb}([a,b])$, *we have*

$$ \bigl\Vert L_{n}\bigl(g',x\bigr) - g'(x) \bigr\Vert _{B} = S^{f}_{\theta}-o \bigl(n^{-\beta}\bigr), $$

*where* $\beta= \min{(\beta_{1}, \beta_{2},\beta_{3})}$.

#### Proof

Proceeding as in the proof of Theorem 2.3, from inequality (2.13), we have

$$\begin{aligned} & \frac{1}{f(h_{r}^{1-\beta})} f\bigl( \bigl\vert \bigl\{ n \in I_{r} : \bigl\Vert L_{n}\bigl(g',x\bigr) - g'(x) \bigr\Vert _{B} \geq\epsilon' \bigr\} \bigr\vert \bigr) \\ &\quad \leq \frac{1}{f(h_{r}^{1-\beta_{1}})}f\biggl( \biggl\vert \biggl\{ n \in I_{r} : \bigl\Vert L_{n}(1, x) - 1 \bigr\Vert _{B} \geq \frac{\epsilon'-\epsilon}{3K}\biggr\} \biggr\vert \biggr)\frac{f(h_{r}^{1-\beta _{1}})}{f(h_{r}^{1-\beta})} \\ &\qquad {}+ \frac{1}{f(h_{r}^{1-\beta_{2}})}f\biggl( \biggl\vert \biggl\{ n \in I_{r}: \bigl\Vert L_{n}(t, x) - x \bigr\Vert _{B} \geq \frac{\epsilon'-\epsilon}{3K}\biggr\} \biggr\vert \biggr)\frac{f(h_{r}^{1-\beta _{2}})}{f(h_{r}^{1-\beta})} \\ &\qquad {}+ \frac{1}{f(h_{r}^{1-\beta_{3}})}f\biggl( \biggl\vert \biggl\{ n \in I_{r} : \bigl\Vert L_{n}\bigl(t^{2}, x\bigr) - x^{2} \bigr\Vert _{B} \geq\frac{\epsilon'-\epsilon}{3K}\biggr\} \biggr\vert \biggr) \frac{f(h_{r}^{1-\beta _{3}})}{f(h_{r}^{1-\beta})} \\ &\quad = \frac{f( \vert \{n \in I_{r} : \Vert L_{n}(1,x) - 1 \Vert _{B} \geq\frac{\epsilon'-\epsilon}{3K} \} \vert )}{f(h_{r}^{1-\beta_{1}})} \biggl(\frac{f(h_{r}^{1-\beta_{1}})}{h_{r}^{1-\beta_{1}}} \biggr) \biggl( \frac {h_{r}^{1-\beta}}{f(h_{r}^{1-\beta})} \biggr) \biggl(\frac{h_{r}^{1-\beta _{1}}}{h_{r}^{1-\beta}} \biggr) \\ &\qquad {}+\frac{f( \vert \{n \in I_{r} : \Vert L_{n}(t,x) - x \Vert _{B} \geq\frac{\epsilon'-\epsilon}{3K} \} \vert )}{f(h_{r}^{1-\beta_{2}})} \biggl(\frac{f(h_{r}^{1-\beta_{2}})}{h_{r}^{1-\beta_{2}}} \biggr) \biggl(\frac {h_{r}^{1-\beta}}{f(h_{r}^{1-\beta})} \biggr) \biggl(\frac{h_{r}^{1-\beta _{2}}}{h_{r}^{1-\beta}} \biggr) \\ &\qquad {}+\frac{f( \vert \{n \in I_{r} : \Vert L_{n}(t^{2},x) - x^{2} \Vert _{B} \geq\frac{\epsilon'-\epsilon}{3K}\} \vert )}{f(h_{r}^{1-\beta_{3}})} \biggl(\frac{f(h_{r}^{1-\beta_{3}})}{h_{r}^{1-\beta_{3}}} \biggr) \biggl(\frac {h_{r}^{1-\beta}}{f(h_{r}^{1-\beta})} \biggr) \biggl(\frac{h_{r}^{1-\beta _{3}}}{h_{r}^{1-\beta}} \biggr). \end{aligned}$$

By using conditions (2.28)–(2.30) and the fact that $\lim_{t \to\infty}\frac{f(t)}{t}>0$, we have

$$ \bigl\Vert L_{n}\bigl(g',x\bigr) - g'(x) \bigr\Vert _{B} = S^{f}_{\theta}-o \bigl(n^{-\beta}\bigr), $$

where $\beta= \min{(\beta_{1}, \beta_{2},\beta_{3})}$. □

## Conclusion

New versions of Korovkin type approximation theorems using the notion of *f*-lacunary statistical convergence have been established. It is shown that any sequence satisfying the conditions of the classical Korovkin first (second) theorem satisfies the conditions of its corresponding *f*-lacunary statistical analog whereas there exists a sequence of positive linear operators which satisfies the conditions of *f*-lacunary statistical analog of Korovkin first (second) theorem without satisfying the conditions of the corresponding classical Korovkin theorem, thereby showing that our results are stronger than the classical ones.

We have also shown that lacunary statistical analog of Korovkin first (second) theorem is stronger than the *f*-lacunary statistical analog of Korovkin first (second) theorem.

Finally, we have characterized those *θ* for which *f*-lacunary statistical analog and the *f*-statistical analog of the Korovkin first (second) theorem are equivalent, of course, under certain restrictions on *f*.
